# Supplementary figures and images for: Sarcopenia increases the risk of post-operative recurrence in patients with non-small cell lung cancer
Source: PLoS One. 2021 Sep 22;16(9):e0257594. doi: 10.1371/journal.pone.0257594 (PMC8457491; doi:10.1371/journal.pone.0257594)

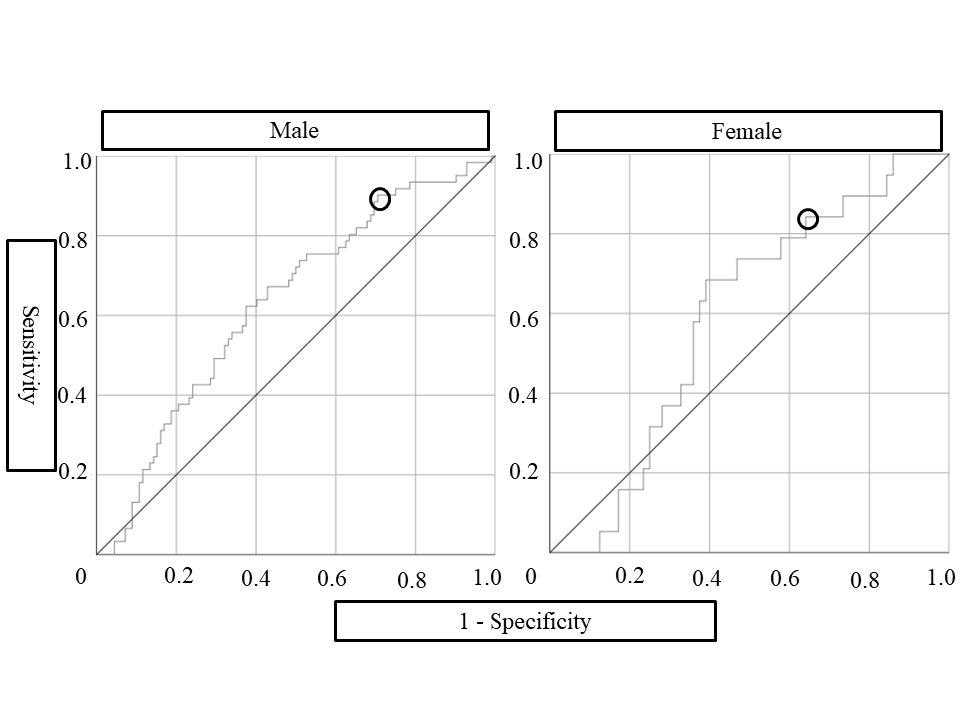

Supplement: S1 Fig — ROC, receiver operating characteristic. (TIF) [file pone.0257594.s001.tif]

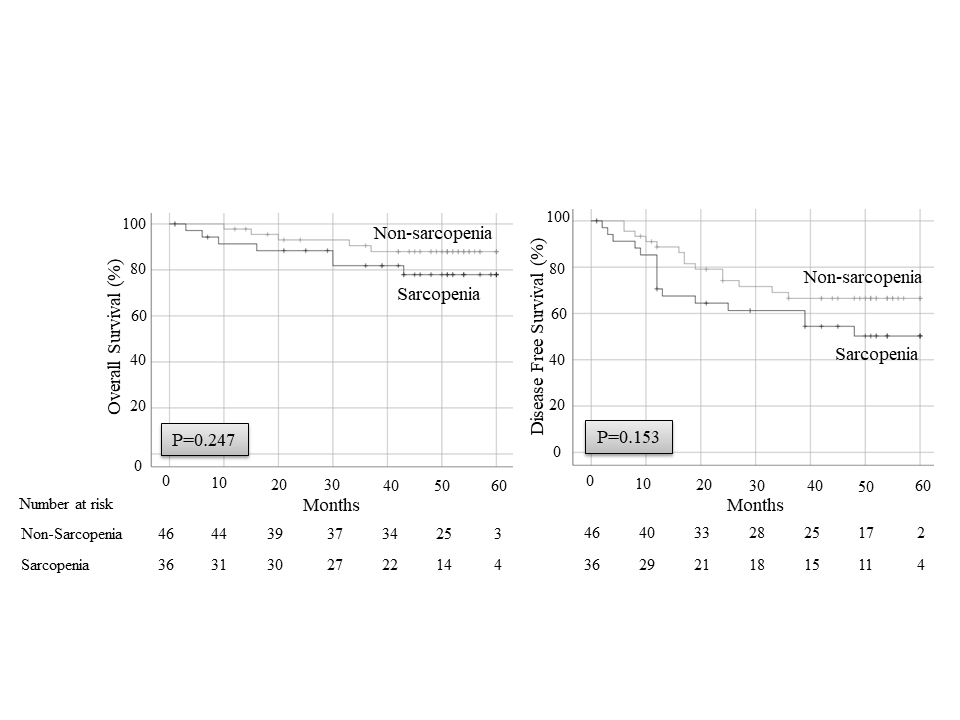

Supplement: S2 Fig — DFS, disease-free survival; OS, overall survival. (TIF) [file pone.0257594.s002.tif]

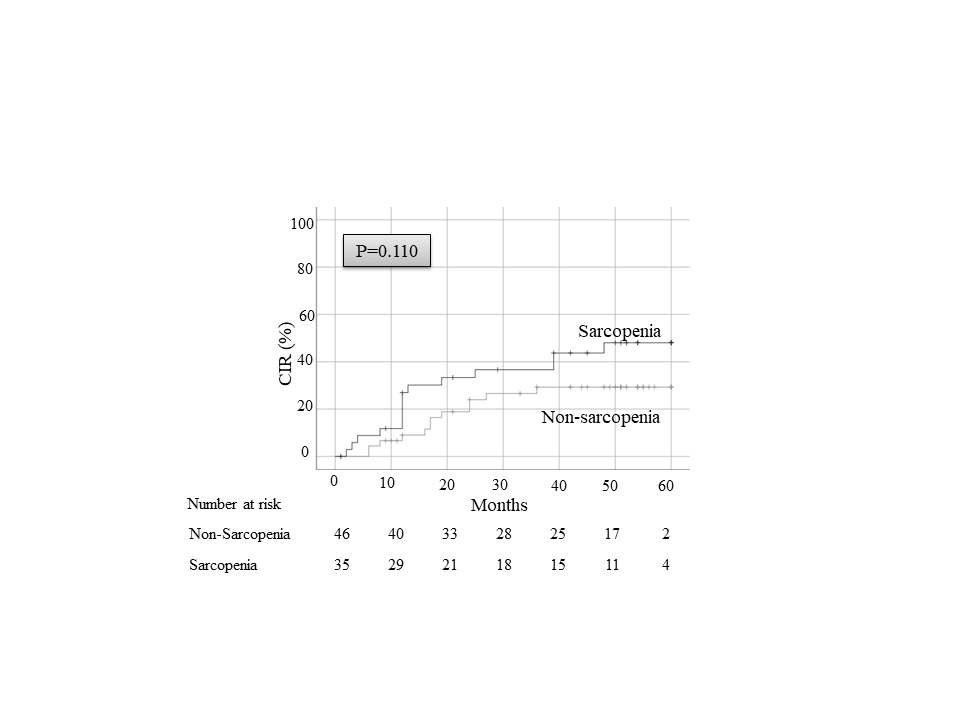

Supplement: S3 Fig — CIR, cumulative incidence of recurrence. (TIF) [file pone.0257594.s003.tif]
