# Supplementary material for: Sarcopenia increases the risk of post-operative recurrence in patients with non-small cell lung cancer
Source: PLoS One. 2021 Sep 22;16(9):e0257594. doi: 10.1371/journal.pone.0257594 (PMC8457491; doi:10.1371/journal.pone.0257594)
Supplement: Attachment — Submitted filename: (R2 subumission) Respons to Reviewers.docx [file pone.0257594.s005.docx]

**Responses to the comments from the Editor and reviewers**

Journal Requirements:

Please review your reference list to ensure that it is complete and correct. If you have cited papers that have been retracted, please include the rationale for doing so in the manuscript text, or remove these references and replace them with relevant current references. Any changes to the reference list should be mentioned in the rebuttal letter that accompanies your revised manuscript. If you need to cite a retracted article, indicate the article’s retracted status in the References list and also include a citation and full reference for the retraction notice.

Response: Thank you for this comment. We have reviewed the reference list and ensured that it is complete. None of the papers cited in our article have been retracted.

Reviewer #1: In this retrospective study, authors have delineated the association between sarcopenia and risk of post-operative recurrence in patients with non-small cell lung cancer. The authors accomplished to address all the comments.

Response: We would like to thank Reviewer #1 for their valuable advice that has helped us improve our manuscript.

Reviewer #2: Manuscipt “Sarcopenia increases the risk of post-operative recurrence in patients with non-small cell lung cancer”

The authors have corrected issues addressed in my initial review as well as issues pointed out by other reviewers.

To make the connection between sarcopenia and recurrence more plausible they have added a validation cohort from patients treated at the same institution but at a different time point. While having a validation cohort is an excellent idea, more could be gained if the cohort would be more independent (other institution, region, country…), but this would make the project much bigger.

They acknowledge the limitations of their study in the discussion section, which is now clearly structured, and provide a revised conclusion taking into account the issue of correlation / causation.

At this point I have no other issues / suggestions regarding this paper.

Response: We would like to thank Reviewer #2 for their pertinent advice that has helped to improve our manuscript.

Reviewer #3: Dear Authors:
Thank you for providing a new version of your manuscript. I have noticed that you addressed major confounding bias by statistical means. This clarifies the interpretation that the reader can have based on your results.
Response: We would like to thank Reviewer #3 for the constructive critique, which has helped improve the manuscript.

I have the following minor concerns to be addressed in the final manuscript if it is to be published:

Response: The revised text in response to the comments from Reviewer #3 is indicated in blue font in the revised manuscript. Our detailed, point-by-point responses to the comments are provided below.

Introduction, line 50: If the statement refers to any evidence published or any own work, please provide reference accordingly.

Response: Thank you for this comment. As suggested, we have included our previously published work under reference [9].

[10] Kawaguchi Y, Hanaoka J, Ohshio Y, Okamoto K, Kaku R, Hayashi K, et al. Does sarcopenia affect postoperative short- and long-term outcomes in patients with lung cancer?-a systematic review and meta-analysis. J Thorac Dis. 2021;13(3):1358-69. doi: 10.21037/jtd-20-3072. PubMed PMID: 33841929; PubMed Central PMCID: PMCPMC8024851. As PLOSOne is not a Clinical cancer exclusive journal, please consider explaining disease burden that makes your research relevant.

Response: Thank you for your advice. We have added some general information on NSCLC and cancer-related sarcopenia to the Introduction.

Page 3, lines 42-45:

“Non-small cell lung cancer (NSCLC) is a major cause of cancer-related deaths globally [1]. Although tumor characteristics, such as histology and TNM stage, and treatment factors, such as surgery and chemotherapy, have traditionally been considered important to determine cancer prognosis, recently, patient factors like sarcopenia have started gaining attention [2]. ”

Methods, lines 101 onwards: please indicate primary variable for statistical analysis according to the objective of the study (line 56) to prevent spurious P-value interpretation.

Response: Thank you for this comment. Accordingly, we have specified that CIR was the primary variable in the Methods section:

Page 6, lines 108-109:

“CIR was defined as the interval between the date of surgery and the date of cancer recurrence (confirmed using an imaging test) and was set as the primary variable.”

Results: Table 1. Please provide characterization of variables to be included in the multivariate analysis, especially Psoas muscle mass index as independent risk factor.

Response: Thank you for this suggestion. We have now provided all variables used in the multivariate analysis, including psoas mass index, in Table 1. Furthermore, following additional analysis, we found that NSCLC patients with sarcopenia can experience more malignant disease than those without sarcopenia. We have added this information to the Discussion of the revised manuscript.

Page 15, lines 202-203:

“In the present study, NSCLC patients with sarcopenia presented with larger tumor size, larger lymph node size, and more vascular invasion than those without sarcopenia.”

Discussion: Exercise is a potential intervention that could affect muscle mass, but it is not solely as should be part of multimodal intervention strategies such as nutritional assessment and treatment. Please discuss if other potential confounders increasing sarcopenia and recurrence risk relationship were not included in your study.

 Response: Thank you for this pertinent comment. Reviewer #1 also mentioned that other potential confounders might increase sarcopenia and cancer recurrence. We have therefore added this information to the section on limitations in the Discussion of the revised manuscript.

Page 17, lines 230-233:

“Second, sarcopenia also occurs in patients suffering from malnutrition, congestive heart failure, chronic obstructive pulmonary disease, chronic renal failure, etc. [23]. Therefore, we cannot exclude the possibility that such potential confounders could have also increased sarcopenia in our patients and influenced cancer recurrence.”
